# Major dietary patterns in relation to menstrual pain: a nested case control study

**DOI:** 10.1186/s12905-018-0558-4

**Published:** 2018-05-21

**Authors:** Nastaran Najafi, Hamidreza Khalkhali, Fatemeh Moghaddam Tabrizi, Rasoul Zarrin

**Affiliations:** 1 Department of Nutrition, School of Medicine, Urmia University of Medical Sciences, Urmia, Iran; 2 Department of Biostatistics and Epidemiology, School of Medicine, Urmia University of Medical Sciences, Urmia, Iran; 3 Department of Midwifery, School of Nursing and Midwifery, Urmia University of Medical Sciences, Urmia, Iran

**Keywords:** Dysmenorrhea, Dietary pattern, Factor analysis

## Abstract

**Background:**

Dysmenorrhea is one of the most prevalent gynecological disorders, experienced by approximately two third of young women during menstruation. According to literature, nutrition can play a key role in the prevalence and severity of dysmenorrhea. This study aims to investigate the relation between dietary patterns and the risk of dysmenorrhea among university students.

**Methods:**

A nested case control study was conducted among 293 students of Urmia University of Medical Sciences who were randomly recruited via a proportional cluster sampling method. From 293 students, 46 students with moderate to severe dysmenorrhea and 54 students without dysmenorrhea were assigned to the case and control groups, respectively. The major dietary patterns of students were identified by factor analysis and the association between dietary patterns and risk of dysmenorrhea was investigated using logistic regression analysis in SPSS 20.

**Results:**

Three major dietary patterns were found and nominated as “Lacto-vegetarian”, “Snacks” and “Mixed food items” patterns. After controlling for family history of dysmenorrhea, subjects in the second and third tertiles of “snacks” pattern had a 4.23 (95% CI = 1.32–13.58, *P* = 0.01) and 3.41 (95% CI = 1.10–10.50, *P* = 0.03) times, respectively, higher chance to experience moderate to severe dysmenorrhea in comparison with subjects in the first tertile. There was no significant association between the risk of dysmenorrhea and two other dietary patterns.

**Conclusions:**

The results indicate that adherence to “snacks” pattern is associated with an increased risk of moderate to severe dysmenorrhea during menstruation among young women.

## Background

Dysmenorrhea is one of the most common gynecologic complaints reported by young women [1]. Dysmenorrhea refers to painful cramps occurring in the lower abdomen or pelvis during menstruation [2] and is experienced by 60–70% of young women [3, 4]. Although dysmenorrhea is not considered a life-threatening disorder, it may reduce quality of life and satisfaction as it can interfere with daily activities as well as familial or social relationships [5]. One of the well-known mechanisms for dysmenorrhea is the elevated release of prostaglandins into the uterine tissue once the menstruation begins. These metabolites increase vasoconstriction and myometrial contractions causing uterine ischemia and pain [1]. In order to relieve menstrual pain, non-steroidal anti-inflammatory drugs (NSAIDs) and oral contraceptive pills (OCPs) are the most preferred medicines used by women [1]. Some potential risk factors of dysmenorrhea are young age (less than 30 years old), body mass index (BMI) less than 20, early menarche, aberrant menstrual flow, longer menstrual cycles, family history of dysmenorrhea and stress [6]. Although the evidence on the relationship between dietary factors and dysmenorrhea is inconclusive, it seems that high consumption of fish, fruits and fiber may reduce the intensity of menstrual pain [7].

To our knowledge, limited studies have investigated the association between dietary patterns and dysmenorrhea. Recently, focusing on dietary pattern approaches has been considered an alternative method to evaluate the association between diet and risk of diseases [8]. Unlike a single-food approach, dietary patterns declare the habitual consumption of individuals according to proportion, frequency and variation of food, drinks and nutrients [9]. Although single-food analyses deliver valuable results, they encounter conceptual and methodological limitations. Since people consume food in the form of meals instead of isolated dietary items, the effect of nutrients and food can synergise or interact with each other, which can hinder the examination of their separate effects [8]. Furthermore, single-food analysis could be confounded by the effect of dietary patterns [8]. In a crossover clinical trial conducted by Barnard et al. [10], an intervention with a low fat vegetarian diet for two menstrual cycles diminished duration and severity of dysmenorrhea. This study aims to examine the relationship between dietary patterns and risk of dysmenorrhea among single and healthy female students of Urmia University of Medical Sciences.

## Methods

### Design and participants

**Fig. 1:**
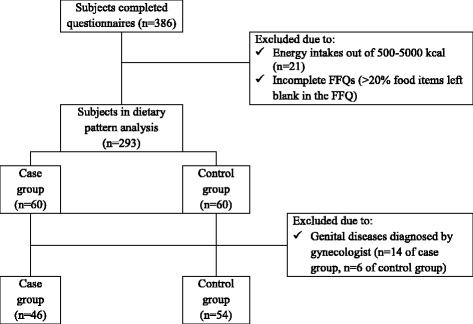
Participant recruitment flow chart

A nested case control study was conducted from April to July 2016 in a medical college located in Urmia, in the West Azarbaijan province of Iran. This University comprises 7 faculties (Medicine, Pharmacy, Paramedical, Health, Nursing and Midwifery, Dentistry and International Branch) and administers 5 teaching hospitals. The sample size was calculated by the following formula:

$$ \mathrm{n}=\frac{\frac{{\mathrm{z}}^2\mathrm{pq}}{{\mathrm{d}}^2}}{1+\frac{1}{\mathrm{N}}\left(\frac{{\mathrm{z}}^2\mathrm{pq}}{{\mathrm{d}}^2}-1\right)} $$

(n = the minimum sample size, N = population size (1972), z = 1.96 for 95% confidence level, p (the prevalence of dysmenorrhea [11]) = 0.74, q = 0.26 and d (the degree of precision) = 0.05). However, taking into account the possible sample loss, a total of 386 samples were estimated. Students were selected by a cluster sampling method. The research team distributed self-administered questionnaires among participants. Students with energy intakes outside the range of 500–5000 kcal/day [12] and those who did not answer more than 20% of Food Frequency Questionnaire (FFQ) items were excluded from the study (*n* = 93) and dietary patterns of 293 students were extracted. Out of 293 students, 60 subjects without dysmenorrhea and 60 subjects with 2nd to 3rd grade dysmenorrhea were randomly assigned to each of control and case groups, respectively. The inclusion criteria in this section was the presence of menstrual periods, being single, not having any chronic diseases, no history of abdominal surgery, no history of alcohol intake and cigarette smoking, no administration of OCPs, dietary supplements and herbal remedies. Body weight and height of both case and control groups were measured and a gynecologist performed abdominal examination and trans-abdominal ultrasonography using an ultrasound scanner (SIUI, CTS-5500, Guangdong, China) to rule out subjects with genital diseases associated with dysmenorrhea (e.g., congenital malformation of the mullerian system, cervical stenosis and ovarian cysts [1]) (*n* = 20). Flow chart of sampling procedure for the study is shown in Fig. 1.

### Data collection

The study questionnaire included five sections. In the first section, students were asked to state their demographic information. In the second section, they were asked about their menstrual characteristics over the last 6 months, including severity of dysmenorrhea, age at menarche, bleeding length, length and regularity of menstrual cycle, severity of menstrual flow and family history of dysmenorrhea. A verbal multidimensional scoring system [13] was used to assess the severity of dysmenorrhea. Based on the pain intensity and its effect on daily activity, symptoms, and the need for analgesics, dysmenorrhea was categorized into four grades: zero (none), 1 (mild), 2 (moderate) or 3 (severe). For the other menstrual characteristics, the questions of the Women’s Health Symptom Survey [14] were used. In the third section, physical activity level was assessed by the short form of International Physical Activity Questionnaire [15, 16]. In this instrument, students were asked to report the frequency and duration of their last 7 days vigorous and moderate activities, walking and sitting. According to the scores, students were categorized into three levels of physical activity: “low” if participants had low activities or did not meet moderate or high category criteria, “moderate” if they had vigorous activities for 3 days or more (at least 20 min per day), a combination of moderate activities and/or walking for 5 days or more (at least 30 min per day) or a combination of walking, moderate or vigorous activities for at least 5 days with MET (Metabolic Equivalent of Task)-minutes/week ≥ 600, and “high” if they had vigorous activities for at least 3 days with MET-minutes/week≥1500 or a combination of walking, moderate or vigorous activities for at least 7 days with MET-minutes/week≥3000. In the fourth section, participants’ depression and anxiety levels over the last 2 weeks were assessed by Patient Health Questionnaire-4 [17]. This instrument had 4 items: the first and second questions measured depression and the third and fourth questions measured anxiety. The response options were “not at all”, “several days”, “more than half days”, and “nearly every day”, which were scored as 0, 1, 2 and 3, respectively. Based on their total scores, students were categorised into four groups: normal (0–2), mild (3–5), moderate (6–7) and severe (9–12). In the fifth section, the participants’ dietary intakes for the last 6 months were assessed by a 115-item semi quantitative FFQ. This questionnaire was adapted from Block design [18] and Tehran Lipid and Glucose Study [19]. The frequency of food intakes options were “2-3 times per day”, “daily”, “4-6 times per week”, “2-3 times per week”, “weekly”, “2-3 times per month”, “monthly”, “1-5 times during the previous 6 month” and “never”. A picture of different portion sizes was given for some food items and for other items, standard household measures (for example “cup” for drinks) or standard units were used. Daily intake of food items (gram/day) was calculated using both frequencies and portion sizes. Total energy intake (kcal/day) of subjects was calculated according to the data available at USDA (United States Department of Agriculture) national nutrient database [20] or other sources [21, 22]. Then, food items were categorized into 30 separate groups based on the previous studies [23, 24] or similarities in their nutrient profile. The FFQ was validated with two 24/h dietary recalls and the correlation coefficients ranged from 0.08 (low fat dairy products) and 0.64 (for high fat dairy products) for food groups.

Body weight was measured (without shoes and in light clothing) to the nearest 0.01 kg using a digital scale (Beurer BF18 Digital Scale). Height was measured in the standing position without shoes to the nearest 0.1 cm. BMI was calculated by dividing weight in kilogram by their height in meters squared.

### Data analysis

All statistical analyses were performed using IBM SPSS Software Package for Windows (version 20.0, Armonk, NY: IBM Corp). The Kolmogorov–Smirnov test was used to determine whether continuous variables follow a normal distribution. Independent-samples t-test, Mann-Whitney U test, Pearson chi squared test and Fisher’s exact test were used to compare continuous or categorical variables between case and control groups. To identify dietary patterns, principal component analysis was carried out and the factors were rotated by varimax rotation. According to factor eigenvalue greater than 1.8, the break point of the scree plot and factor interpretability, factors (dietary patterns) were noted. These factors were nominated based on food groups with factor loading> 0.2. After computing factor *scores* for each individual, we categorized participants by tertiles of scores and the first tertile was considered as a reference. We used logistic regression to calculate the crude and adjusted odds ratios (OR) and the 95% confidence intervals to interpret the association between dietary patterns and risk of dysmenorrhea. Adjusted ORs were calculated by adjusting for family history of dysmenorrhea. A *P* value of < 0.05 was considered statistically significant.

## Results

Among 293 students, the frequency of dysmenorrhea was 74.3% (95% CI; 69.3, 79.3%) (Mild dysmenorrhea was 17.7%, moderate dysmenorrhea was 45.7% and severe dysmenorrhea was 10.9%). The mean ± SD (Standard Deviation) values for age of participants was 22.2 ± 2.18 years (range, 19–30), age at menarche was 13.23 ± 1.34 years (range, 9–18), length of menstrual cycle was 28.39 ± 3.45 days (range, 20–50) and bleeding length was 5.6 ± 1.47 days (range, 2–11). As for marital and residential status, of the 293 students, 266 (90.8%) were single (9.2% were married) and 165 (54.3%) resided at the dormitories. The menstrual characteristics, physical activity and depression/anxiety levels of 46 cases and 54 controls are shown in Table 1. Compared to the control group, cases tended to have higher levels of menstrual flow (*F* = 5.89, *P* = 0.01), higher frequency of family history of dysmenorrhea (Pearson chi squared = 12.4, df = 1, *P* < 0.001) and higher levels of depression and anxiety (Pearson chi squared = 7.92, df = 3, *P* = 0.02). No significant difference was found in other characteristics between case and control groups.

**Table 1:** Characteristics of the case and control groups

Variables	Cases (*n* = 46)	Controls (*n* = 54)	*P*-value^a^
Age (y) (Mean ± SD)	21.89 ± 1.43	21.92 ± 1.83	0.41
Age at menarche (y) (Mean ± SD)	13.43 ± 1.47	13.61 ± 1.43	0.28
Length of menstrual cycle (days)	28.63 ± 1.92	28.44 ± 3.23	0.19
Menstrual cycle regularity, n (%)			0.49
Yes	46 (46.9)	52 (53.1)	
No	0	2 (100)	
Bleeding length (days)	5.52 ± 1.5	5.19 ± 1.1	0.09
Severity of menstrual flow, n (%)			0.01
Low	0	3 (100)	
Moderate	30 (41.7)	42 (58.3)	
Severe	16 (64)	9 (36)	
Family history of dysmenorrhea, n (%)			< 0.001
Yes	41 (56.9)	31 (43.1)	
No	5 (17.9)	23 (82.1)	
Physical activity level, n (%)			0.71
Low	11 (47.8)	12 (52.2)	
Moderate	27 (48.2)	29 (51.8)	
High	8 (38.1)	13 (61.9)	
Depression-anxiety levels, n (%)			0.02
Normal	9 (30)	21 (70)	
Mild	24 (49)	25 (51)	
Moderate	8 (80)	2 (20)	
Severe	5 (45.5)	6 (54.5)	
BMI (kg/m^2^), (Mean ± SD)	21.71 ± 2.69	21.59 ± 2.39	0.4
Daily energy intake (Kcal), (Mean ± SD)	2655.20 ± 822.39	2706.99 ± 892.50	0.59

^a^Significance is derived from Pearson chi squared test for categorical variables (family history of dysmenorrhea, physical activity level and depression-anxiety level); fisher’s exact test for severity of menstrual flow and cycle regularity; independent-samples t-test for BMI and daily energy intake and Mann-Whitney U test for age, age at menarche, length of menstrual cycle and bleeding length

Food items in the FFQ were summarized in 30 groups shown in Table 2. Adequacy of sample size and suitability of data for applying factor analysis were confirmed by Kaiser-Meyer-Olkin (KMO = 0.603) and Bartlett’s tests (*P* < 0.001). Three major patterns were derived which explained 23.65% of total variance. Factor loading values of food groups for dietary patterns are shown in Table 3. “Lacto-vegetarian” pattern, explained 10.65% of total variance, included high intakes of vegetables (all kinds), legumes, fruits, dairy products, vegetable patties (an Iranian egg-based food), nuts, pickles and butter. “Snacks” pattern explained 6.76% of variance and had high factor loadings on sugars, salty snacks, sweets and desserts, tea and coffee, salt, fruit juices and added fat. “Mixed food items” pattern explained the least variance (6.23%) and included high intakes of poultry, mayonnaise, sugar sweetened beverages, fast food, potatoes, egg and red meats.

**Table 2:** Food groups used in factor analysis

Food groups	Food items
Red meats	Red meats, ground meats, meatballs, kabob, heart or liver (organ meats)
Fish	Fish, canned tuna fish
Poultry	Chicken (with or without skin)
Eggs	Eggs
Low fat dairy products	Low fat milk, low fat yoghurt, dough (yoghurt drink)
High fat dairy products	Whole milk, chocolate milk, high fat yoghurt, cream cheese, other cheese, ice cream, cream
Tea and coffee	Tea, coffee
Fruit	Apples or pears, strawberries, cherries, apricots, grapes, peaches or nectarines, figs, cantaloupe or watermelon, bananas, mangos, persimmons, pomegranates, kiwis, oranges or tangerines or lemons, grapefruit, canned fruits, dates, raisins, dried fruits
Fruit juices	Fruit juices, fruit nectars
Sugar sweetened beverages	All kinds of sugar sweetened drinks, carbonated beverages
Cruciferous vegetables	Cabbage
Green leafy vegetables	Spinach, lettuce
Yellow vegetables	Carrots
Tomatoes	Tomatoes, tomato sauce
Other Vegetables	Green herbs, cucumber, squash or eggplant, celery, mushrooms, garlic, onion, radish, peppers, olives, corn, green beans
Vegetable patties	Green Herbs patty, potato patty, green bean patty
Legumes	Chickpeas, lentils, beans, peas, soybeans
Nuts	Walnuts, almonds, other nuts, Seeds
Sweets and desserts	Puddings, cakes, cookies, cream cakes or doughnuts, biscuits or wafers
Pickles	Cucumber pickle, other pickles
Butter	Butter
Sugars	Sugars, chocolates, jelly, honey or jam, candies
Salt	Table salt
Grains	Iranian breads (lavash, sangak or barbari), rice, pasta, barley
Potatoes	Potatoes
French fries	French fries
Fast food	Pizza, burgers, chicken sandwiches, fried chicken, sausages, lunch meats
Salty snacks	Potato Chips, corn puffs, crackers, popcorn
Mayonnaise	Mayonnaise
Added fats	Added fats

**Table 3:** Factor loadings^a^ of food groups in derived dietary patterns

Food groups	Dietary patterns		
	Lacto-vegetarian	Snacks	Mixed food items
Other Vegetables	0.641		
Tomatoes	0.565		
Yellow vegetables	0.534		
Legumes	0.523		
Fruit	0.502		
Green leafy vegetables	0.443		
Cruciferous vegetables	0.424		
Low fat dairy products	0.403		
Vegetable patties	0.393		
High fat dairy products	0.364		0.287
Nuts	0.297	0.244	
Pickles	0.275		
Butter	0.246		
Sugars		0.694	
Salty snacks		0.654	
Sweets and desserts	0.217	0.539	
Tea and coffee		0.469	
Salt	0.292	0.456	
Fruit juices		0.338	0.226
Added fats		0.330	
Poultry			0.637
Mayonnaise			0.598
Sugar sweetened beverages			0.570
Fast food		0.382	0.441
Potatoes			0.420
Eggs			0.382
Red meats			0.301
French fries	-	-	-
Fish	**-**	**-**	**-**
Grains	**-**	**-**	**-**
Percent of variance explained	10.65	6.76	6.23

^a^Factor loadings < 0.2 are omitted for simplicity

Crude odds ratios for risk of dysmenorrhea across the tertiles of derived dietary patterns are presented in Table 4. Subjects in the second and third tertiles of the “snacks” pattern, had a 4.07 (95% CI: 1.35–12.22, *P* = 0.01) and 3.15 (95% CI: 1.09–9.12, *P* = 0.03) times, respectively, greater chance to experience 2nd to 3rd grade dysmenorrhea in comparison with subjects in the first tertile. There was no significant association between dysmenorrhea and the other two patterns. Adjusted odds ratios for dysmenorrhea risk across the tertiles of “snacks” pattern are shown in Table 5. After controlling for family history of dysmenorrhea, the results for “snacks” pattern stayed relatively unchanged and subjects in the second and third tertiles were 4.23 (95% CI: 1.32–13.58, *P* = 0.01) and 3.41 (95% CI: 1.10–10.50, *P* = 0.03) times, respectively, more likely to experience high grade dysmenorrhea when compared to subjects in the first tertile.

**Table 4:** Crude odds ratios for dysmenorrhea risk across tertiles of three major dietary patterns

Dietary patterns	Β (SE)^a^	OR^b^	(95% CI^c^)	*P* ^d^
Lacto-vegetarian				0.47
T1	–	1	–	–
T2	−0.47	0.62	(0.25–1.56)	0.31
T3	−0.58	0.55	(0.19–1.59)	0.27
Snacks				0.03
T1	–	1	–	–
T2	1.40	4.07	(1.35–12.22)	0.01
T3	1.15	3.15	(1.09–9.12)	0.03
Mixed food items				0.21
T1	–	1	–	–
T2	−0.17	0.84	(0.39–2.86)	0.73
T3	−0.82	0.43	(0.27–1.90)	0.09

^a^Regression coefficient

^b^Odds ratio

^c^Confidence interval

^d^No potential risk factor was adjusted

**Table 5:** Adjusted odds ratios for dysmenorrhea risk across tertiles of “snacks” pattern

Dietary patterns	Β (SE)^a^	OR^b^	(95% CI^c^)	*P* ^d^
Snacks				0.03
T1	–	1	–	–
T2	1.44	4.23	(1.32–13.58)	0.01
T3	1.22	3.41	(1.10–10.50)	0.03

^a^Regression coefficient

^b^Odds ratio

^c^Confidence interval

^d^Adjusted for family history of dysmenorrhea

## Discussion

In the present study, three major dietary patterns including “lacto-vegetarian”, “snacks” and “mixed food items” were derived using factor analysis. The three patterns explained 23.65% of total variance. The “snacks” pattern was associated with risk of dysmenorrhea before and after controlling for family history of dysmenorrhea. According to a provocative commentary by Anderson and Patterson [25], unhealthy snack food (called “junk food”) provide suboptimal nutrition and excessive energy, fat, sugar and sodium. Furthermore, over-consumption of these kinds of food, decreases the intakes of nutrient-dense food which results in low intakes and low serum concentrations of most micronutrients, e.g., vitamin E, B6 and calcium [26]. A Cochrane review of several clinical trials which summarized the efficacy and safety of dietary interventions for the treatment of dysmenorrhea, have explained the effectiveness of such micronutrients in reducing the duration and intensity of dysmenorrhea [27]. The current study found no association between “lacto-vegetarian” and risk of dysmenorrhea. This result was inconsistent with the study conducted by Barnard et al. [10] who reported that intervention by a low fat vegetarian diet for two menstrual cycles can reduce the intensity of dysmenorrhea in a sample of 33 women aged at least 18 years old. The diet in the mentioned study included high intakes of cereals, legumes, vegetables and fruits with restriction on animal products, oils, fried food, avocados, olives, nuts, nut butters and seeds. Since the “lacto-vegetarian” pattern included high-fat dairy products, nuts and butter, it could not be considered a low fat diet. In the mentioned study, both single and married women with a wide range of ages (> 18 years) were included to study. Moreover, they did not adjust their analyses for potential cofounders and also did not indicate any exploration of the pathologic diseases of dysmenorrhea through medical examinations. Applying different pain measuring instruments can also explain different findings in both studies. This study has some limitations. First, although ultrasonography was applied to rule out participants with certain underlying genital diseases, this method was not accurate enough to diagnose all of the related diseases. For instance, The gold standard for the diagnosis of endometriosis is laparoscopy [28] which was not applicable in the current study. Hence, discrimination between primary and secondary dysmenorrhea was impossible and menstrual pain as a whole was considered. Second, no additional data about the type of analgesics and their usage pattern during menstruation was obtained from subjects. Third, we evaluated the dietary intakes of individuals using FFQs which increased the possibility of measurement error. Forth, dietary pattern approaches were prone to subjective decisions in the fields of food categorization, selection or nomination of factors which made it difficult to compare the patterns derived by different studies.

## Conclusions

The results of this research imply that diet, characterized by a high consumption of sugars, salty snacks, sweets and desserts, tea and coffee, salt, fruit juices and added fat (labeled as “snacks” pattern), is associated with an increased risk of dysmenorrhea among young women. More research is needed in this field to optimize the dietary patterns of individuals suffering from this problem.

## Abbreviations

BMI: Body Mass Index
CI: Confidence Interval
FFQ: Food Frequency Questionnaire
KMO: Kaiser-Meyer-Olkin
MET: Metabolic Equivalent of Task
NSAID: Non-Steroidal Anti-Inflammatory Drug
OCP: Oral Contraceptive Pill
OR: Odds Ratio
SD: Standard Deviation
USDA: United States Department of Agriculture
